# Orientational disorder in the one-dimensional coordination polymer *catena*-poly[[bis­(acetyl­acetonato-κ^2^ *O*,*O*′)cobalt(II)]-μ-1,4-di­aza­bicyclo­[2.2.2]octane-κ^2^ *N* ^1^:*N* ^4^]

**DOI:** 10.1107/S205698901600428X

**Published:** 2016-03-31

**Authors:** Florina Dumitru, Ulli Englert, Beatrice Braun

**Affiliations:** a Faculty of Applied Chemistry and Materials Science, University Politehnica of Bucharest, Polizu 1, RO-011061 Bucharest, Romania; b Institute of Inorganic Chemistry, RWTH Aachen University, Landoltweg 1, 52074 Aachen, Germany; c Institute of Chemistry, Humboldt University of Berlin, Brook-Taylor-Strasse 2, D-12489 Berlin, Germany

**Keywords:** crystal structure, one-dimensional coordination polymer, orientational disorder, acetyl­acetonate complexes, cobalt(II), DABCO, magnetic behaviour

## Abstract

The title compound, [Co(C_5_H_7_O_2_)_2_(C_6_H_12_N_2_)]_*n*_, was obtained as a one-dimensional coordination polymer from bis­(acetyl­acetonato)di­aqua­cobalt(II), [Co(acac)_2_(OH_2_)_2_], and 1,4-di­aza­bicyclo­[2.2.2]octane (DABCO), a di­amine with good bridging ability and rod-like spacer function. In the chain complex that extends along the *c* axis, the Co^II^ atom is six-coordinated, the O-donor atoms of the chelating acac ligands occupying the equatorial positions and the bridging DABCO ligands being in *trans*-axial positions. In the crystal structure, the DABCO ligand is conformationally disordered in a 50:50 manner as a result of its location across a crystallographic mirror plane. The metal–metal distance is very close to that in a related compound exhibiting weak anti­ferromagnetic exchange between the Co^II^ ions, and the title compound can thus be useful for obtaining more information about the contribution of different bridges to the magnetic coupling between paramagnetic ions.

## Abstract 2

The one-dimensional coordination polymer, self-assembled from bis­(acetyl­acetonato)cobalt(II) units as metal–complex *connectors* and 1,4-di­aza­bicyclo­[2.2.2]octane (DABCO) as *linkers*, can serve for a comparative investigation of the magnetic behaviour of analogous compounds. Space filling more symmetric than atom positions leads to pronounced orientational disorder for the DABCO ligand.

## Chemical context   

Self-assembly of coordination polymers from simple building blocks is of considerable inter­est due to their diverse architectures and potential applications in catalysis and advanced materials, such as magnetic, optic and electronic materials.
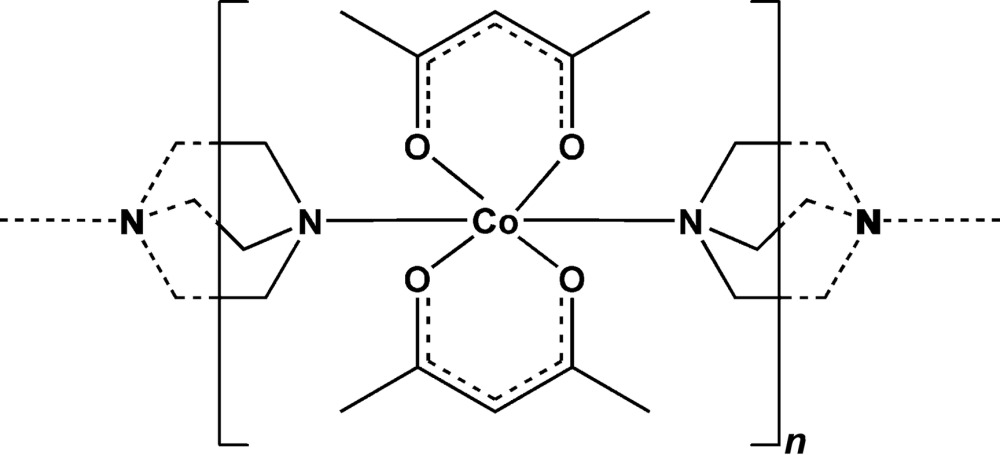


In this paper, two simple building blocks, namely 1,4-di­aza­bicyclo­[2.2.2]octane (DABCO), a di­amine with good bridging ability and rod-like spacer function, and the unsatur­ated square-planar metal complex bis­(acetyl­aceto­nato-κ^2^ *O*,*O*′)cobalt(II), [Co(acac)_2_], have been chosen to design a one-dimensional coordination polymer in which the paramagnetic Co^II^ ions are separated by a distance of 7.2328 (7) Å. This metal–metal distance is very close to the distance of 7.267 (3) Å reported by Ma *et al.* (2001 ▸) for the structurally related [Co(acac)_2_(pyrazine)]_*n*_ compound which exhibits weak anti­ferromagnetic exchange between the Co^II^ ions.

Within this context, the title compound *catena*-poly[[bis(acetyl­acetonato-κ^2^ *O*,*O*′)cobalt(II)]-μ-1,4-di­aza­bicyclo­[2.2.2]octane-κ^2^ *N* ^1^:*N* ^4^], [Co(acac)_2_(DABCO)]_*n*_, (I), can serve for a comparative investigation of the magnetic behaviour of analogous compounds and, thus, allow more information about the contribution of different bridges to the magnetic coupling between paramagnetic ions to be obtained.

## Structural commentary   

In the crystalline state, the title compound, (I), represents a one-dimensional coordination polymer self-assembled from bis­(acetyl­acetonato)cobalt(II) units as metal–complex *connectors* and 1,4-di­aza­bicyclo­[2.2.2]octane (DABCO) as *linkers*.

The acetyl­acetonate (acac) ligand, which is the deproton­ated form of acetyl­acetone (pentane-2,4-dione, acacH), is a well-known mononegative *O*,*O*′-chelating donor agent and its metal coordination chemistry is well documented [for reviews on the coordination chemistry of acac ligands, see: Aromí *et al.* (2008 ▸); Bray *et al.* (2007 ▸); Vigato *et al.* (2009 ▸)]. For DABCO, the bridging coordination behaviour is most exploited for the generation of coordination polymers and metal–organic frameworks (MOFs), with Zn^2+^ being the most common metal ion used in these structures [for representative examples, see: Furukawa *et al.* (2009 ▸); Uemura *et al.* (2007 ▸)].

**Figure 1:**
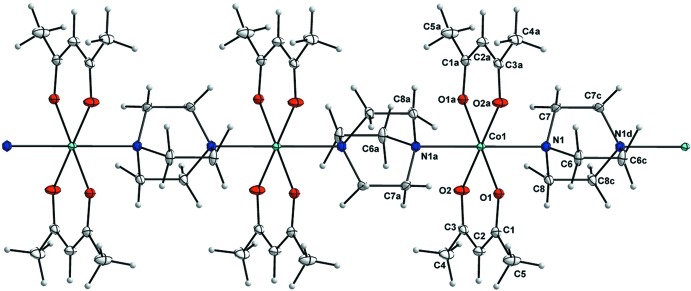
A section of the coordination polymer of (I). Only one of the 50:50 DABCO disorder forms and one orientation of the disordered acac methyl groups are shown. Displacement ellipsoids are drawn at the 50% probability level and H atoms are represented by circles. [Symmetry codes: (*a*) −*x*, −*y*, −*z*; (*c*) *x*, *y*, −*z* + 1; (*d*) −*x*, −*y*, −*z* + 1.]

The complex crystallizes in the ortho­rhom­bic *Pnnm* space group with the metal atom on a special position with site symmetry ..2/*m*. The Co^II^ atom shows an octa­hedral environment defined by four equatorial acac O atoms on a mirror plane, with bond lengths ranging from 2.0299 (10) to 2.0411 (10) Å, and with two N atoms of bridging DABCO groups on a twofold rotation axis in the axial positions at distances of 2.3071 (12) Å (Fig. 1 ▸).

## Supra­molecular features   

**Figure 2:**
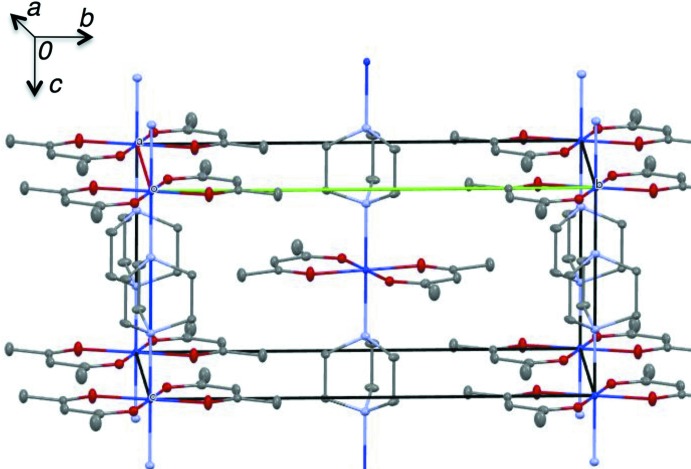
The mol­ecular packing of the coordination polymer chains.

The centrosymmetric DABCO ligand is bonded to two [Co(acac)_2_] units, which gives rise to the formation of chains extending along the *c* axis (Fig. 2 ▸). The individual chains run parallel in the crystal and do not inter­act with each other. This polymer is essentially a one-dimensional coordination polymer, the only structural motif that is present being based on the Co^II^ coordination requirements.

## Database survey   

Although some polymeric complexes of the form [Co(acac)_2_(μ-di­amine)]_*n*_ [di­amine = NH_2_–*R*–NH_2_, with *R* = C_*y*_H_2*y*+1_ (*y* = 6, 11, 12; Fine, 1973 ▸), piperazine (Pellacani *et al.*, 1973 ▸), 2,5-di­methyl­pyrazine (Blake & Hatfield, 1978 ▸), and 1,2-bis­(4-pyrid­yl)ethane and *trans*-1,2-bis­(4-pyrid­yl)ethyl­ene (Atienza *et al.*, 2008 ▸)] have been synthesized over the years, their structures were elucidated only on the basis of spectroscopic and magnetic analyses. [Co(acac)_2_(μ-di­amine)]_*n*_ complexes similar in structure to the title compound, with square-planar [Co(acac)_2_] units connected by bridging di­amine ligands into infinite linear chains, were retrieved from the Cambridge Structural Database (CSD, Version 5.36 of November 2014; Groom & Allen, 2014 ▸), *viz.* [Co(acac)_2_{μ-1,3-bis­(pyridin-4-yl)propane}]_*n*_ (Lennartson & Håkansson, 2009 ▸), [Co(acac)_2_(pyrazine)]_*n*_ and [Co(acac)_2_(4,4′-bi­pyridine)]_*n*_ (Ma *et al.*, 2001 ▸).

## Synthesis and crystallization   

[Co(acac)_2_(H_2_O)_2_] was prepared by precipitation of CoCl_2_·6H_2_O with aqueous ammonia, followed by solubilization and complexation with acetyl­acetone. Elemental analysis calculated for [Co(C_5_H_7_O_2_)_2_(H_2_O)_2_] (%): C 40.96, H 6.14; found: C 40.94, H 6.19.

[Co(acac)_2_(H_2_O)_2_] (293 mg, 1 mmol) and 1,4-di­aza­bicyclo­[2.2.2]octane (DABCO) (112 mg, 1 mmol) were stirred in CH_3_OH (15 ml) at 333 K for 1 h. The pink precipitate which formed was collected by filtration and redissolved in dimethyl sulfoxide (DMSO, 5 ml). Elemental analysis calculated for [Co(acac)_2_(DABCO)] (%): C 52.04, H 7.05, N 7.59; found: C 51.63, H 7.39, N 7.41. Layering the solution of the complex in DMSO with CH_3_OH at 293 K gave pale-pink crystals suitable for X-ray single-crystal analysis.

Elemental analyses were carried out on a Heraeus CHNO Rapid apparatus (Institute of Inorganic Chemistry, RWTH Aachen University).

## Refinement   

**Figure 3:**
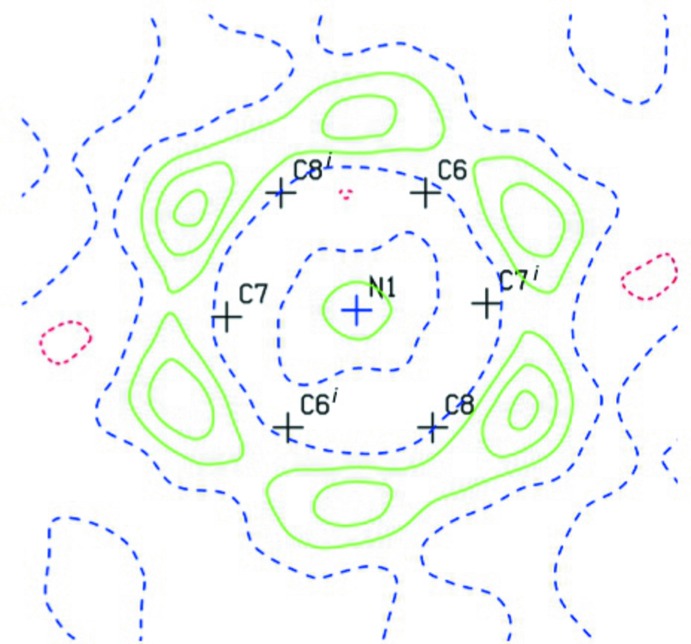
Difference-density Fourier synthesis in the *ab* plane through three DABCO C atoms before assignment of the DABCO H-atom positions; contour lines are drawn at 0.2 e A^−3^ inter­vals.

Crystal data, data collection and structure refinement details are summarized in Table 1 ▸. Space filling more symmetric than atom positions leads to pronounced orientational disorder (Herberich *et al.*, 1993 ▸) for the DABCO ligand over two positions due to mirror symmetry. As a result, the site occupancies of the C atoms are constrained to 0.5. In principle, the same should be true for the associated H atoms, their alternative positions for the different C positions overlap very closely, thus forming the hexa­gon of local residual electron-density maxima about the C-atom scaffold shown in Fig. 3 ▸. These maxima can be freely refined as H atoms with reasonable C—H geometry and displacement parameters.

H atoms attached to C atoms were calculated, introduced in their idealized positions and treated as riding, with C—H = 0.95 Å and *U*_iso_(H) = 1.5*U*_eq_(C) for methyl H atoms and *U*_iso_(H) = 1.2*U*_eq_(C) otherwise. For consistency, we opted to calculate the positions of the DABCO H atoms and fix them in their idealized positions. Due to the fact that the acac ligand lies on a mirror plane, the acac methyl groups are therefore equally disordered over two orientations.

## Supplementary Material

Crystal structure: contains datablock(s) I, global. DOI: 10.1107/S205698901600428X/wm5278sup1.cif

Structure factors: contains datablock(s) I. DOI: 10.1107/S205698901600428X/wm5278Isup2.hkl

CCDC reference: 1454848

Additional supporting information:  crystallographic information; 3D view; checkCIF report

## Figures and Tables

**Table 1:** Experimental details

Crystal data	
Chemical formula	[Co(C_5_H_7_O_2_)_2_(C_6_H_12_N_2_)]
*M*_r_	369.32
Crystal system, space group	Orthorhombic, *P* *n* *n* *m*
Temperature (K)	100
*a*, *b*, *c* (Å)	7.7468 (3), 15.1573 (4), 7.2328 (7)
*V* (Å^3^)	849.28 (9)
*Z*	2
Radiation type	Mo *K*α
μ (mm^−1^)	1.03
Crystal size (mm)	0.48 × 0.10 × 0.04

Data collection	
Diffractometer	Stoe *IPDS* 2T
Absorption correction	Multi-scan (*MULABS* in *PLATON*; Spek, 2003 ▸)
*T*_min_, *T*_max_	0.637, 0.960
No. of measured, independent and observed [*I* > 2σ(*I*)] reflections	11457, 1045, 944
*R*_int_	0.045
(sin θ/λ)_max_ (Å^−1^)	0.649

Refinement	
*R*[*F* ^2^ > 2σ(*F* ^2^)], *wR*(*F* ^2^), *S*	0.020, 0.055, 1.09
No. of reflections	1045
No. of parameters	81
H-atom treatment	H-atom parameters constrained
Δρ_max_, Δρ_min_ (e Å^−3^)	0.25, −0.39

Computer programs: *X-AREA* (Stoe & Cie, 2002 ▸), *X-RED* (Stoe & Cie, 2002 ▸), *SHELXS2013* (Sheldrick, 2008 ▸), *SHELXL2014* (Sheldrick, 2015 ▸), *DIAMOND* (Brandenburg, 1999 ▸), *Mercury* (Macrae *et al.*, 2006 ▸), *PLATON* (Spek, 2009 ▸) and *publCIF* (Westrip, 2010 ▸).

## supplementary crystallographic information

### Crystal data

**Table:**

[Co(C_5_H_7_O_2_)_2_(C_6_H_12_N_2_)]	*D*_x_ = 1.444 Mg m^−^^3^
*M**_r_* = 369.32	Mo *K*α radiation, λ = 0.71073 Å
Orthorhombic, *P**n**n**m*	Cell parameters from 16455 reflections
*a* = 7.7468 (3) Å	θ = 3.8–29.5°
*b* = 15.1573 (4) Å	µ = 1.03 mm^−^^1^
*c* = 7.2328 (7) Å	*T* = 100 K
*V* = 849.28 (9) Å^3^	Elongated plate, pale pink
*Z* = 2	0.48 × 0.10 × 0.04 mm
*F*(000) = 390	

### Data collection

**Table:**

Stoe IPDS 2T diffractometer	1045 independent reflections
Radiation source: sealed X-ray tube, 12 x 0.4 mm long-fine focus	944 reflections with *I* > 2σ(*I*)
Plane graphite monochromator	*R*_int_ = 0.045
Detector resolution: 6.67 pixels mm^-1^	θ_max_ = 27.5°, θ_min_ = 3.8°
rotation method scans	*h* = −10→10
Absorption correction: multi-scan (*MULABS* in *PLATON*; Spek, 2003)	*k* = −19→19
*T*_min_ = 0.637, *T*_max_ = 0.960	*l* = −8→9
11457 measured reflections	

### Refinement

**Table:**

Refinement on *F*^2^	0 restraints
Least-squares matrix: full	Hydrogen site location: inferred from neighbouring sites
*R*[*F*^2^ > 2σ(*F*^2^)] = 0.020	H-atom parameters constrained
*wR*(*F*^2^) = 0.055	*w* = 1/[σ^2^(*F*_o_^2^) + (0.0389*P*)^2^] where *P* = (*F*_o_^2^ + 2*F*_c_^2^)/3
*S* = 1.09	(Δ/σ)_max_ < 0.001
1045 reflections	Δρ_max_ = 0.25 e Å^−^^3^
81 parameters	Δρ_min_ = −0.39 e Å^−^^3^

### Special details

**Table:**

Geometry. All e.s.d.'s (except the e.s.d. in the dihedral angle between two l.s. planes) are estimated using the full covariance matrix. The cell e.s.d.'s are taken into account individually in the estimation of e.s.d.'s in distances, angles and torsion angles; correlations between e.s.d.'s in cell parameters are only used when they are defined by crystal symmetry. An approximate (isotropic) treatment of cell e.s.d.'s is used for estimating e.s.d.'s involving l.s. planes.

### Fractional atomic coordinates and isotropic or equivalent isotropic displacement parameters (Å^2^)

**Table:**

	*x*	*y*	*z*	*U*_iso_*/*U*_eq_	Occ. (<1)
Co1	0.0000	0.0000	0.0000	0.00790 (10)	
O1	0.24789 (12)	0.04561 (6)	0.0000	0.0122 (2)	
O2	−0.08057 (13)	0.12744 (7)	0.0000	0.0173 (2)	
N1	0.0000	0.0000	0.31898 (17)	0.0106 (3)	
C1	0.29942 (18)	0.12455 (10)	0.0000	0.0134 (3)	
C2	0.19083 (19)	0.19922 (9)	0.0000	0.0148 (3)	
H2	0.2442	0.2557	0.0000	0.018*	
C3	0.01048 (18)	0.19635 (10)	0.0000	0.0127 (3)	
C4	−0.0887 (2)	0.28217 (9)	0.0000	0.0196 (3)	
H4A	−0.1733	0.2817	0.1008	0.029*	0.5
H4B	−0.1488	0.2891	−0.1184	0.029*	0.5
H4C	−0.0085	0.3314	0.0176	0.029*	0.5
C5	0.49196 (18)	0.13858 (12)	0.0000	0.0228 (3)	
H5A	0.5190	0.1944	−0.0619	0.034*	0.5
H5B	0.5481	0.0899	−0.0659	0.034*	0.5
H5C	0.5340	0.1405	0.1277	0.034*	0.5
C6	0.1745 (3)	−0.02067 (16)	0.3932 (3)	0.0167 (4)	0.5
H6A	0.2582	0.0237	0.3477	0.020*	0.5
H6B	0.2115	−0.0793	0.3477	0.020*	0.5
C7	−0.1237 (3)	−0.06034 (13)	0.3934 (3)	0.0155 (4)	0.5
H7A	−0.0975	−0.1206	0.3484	0.019*	0.5
H7B	−0.2402	−0.0442	0.3484	0.019*	0.5
C8	−0.0438 (3)	0.09127 (13)	0.3934 (3)	0.0142 (4)	0.5
H8A	−0.1589	0.1093	0.3477	0.017*	0.5
H8B	0.0420	0.1345	0.3477	0.017*	0.5

### Atomic displacement parameters (Å^2^)

**Table:**

	*U*^11^	*U*^22^	*U*^33^	*U*^12^	*U*^13^	*U*^23^
Co1	0.01008 (15)	0.00601 (15)	0.00760 (15)	−0.00078 (8)	0.000	0.000
O1	0.0133 (5)	0.0109 (5)	0.0122 (5)	−0.0013 (4)	0.000	0.000
O2	0.0147 (5)	0.0089 (5)	0.0282 (6)	−0.0001 (4)	0.000	0.000
N1	0.0122 (6)	0.0114 (6)	0.0081 (5)	0.0000 (4)	0.000	0.000
C1	0.0158 (7)	0.0143 (7)	0.0102 (6)	−0.0041 (5)	0.000	0.000
C2	0.0195 (7)	0.0093 (6)	0.0156 (6)	−0.0036 (5)	0.000	0.000
C3	0.0202 (7)	0.0089 (7)	0.0089 (6)	0.0004 (5)	0.000	0.000
C4	0.0238 (8)	0.0106 (6)	0.0243 (8)	0.0022 (5)	0.000	0.000
C5	0.0151 (7)	0.0177 (8)	0.0355 (9)	−0.0031 (5)	0.000	0.000
C6	0.0143 (9)	0.0269 (10)	0.0090 (9)	0.0046 (8)	0.0003 (8)	0.0007 (8)
C7	0.0219 (10)	0.0169 (9)	0.0079 (9)	−0.0109 (8)	0.0005 (8)	−0.0015 (7)
C8	0.0252 (10)	0.0099 (9)	0.0075 (9)	0.0030 (7)	−0.0007 (8)	0.0006 (7)

### Geometric parameters (Å, º)

**Table:**

Co1—O2^i^	2.0299 (10)	C2—H2	0.9500
Co1—O2	2.0299 (10)	C3—C4	1.511 (2)
Co1—O1	2.0410 (10)	C4—H4A	0.9800
Co1—O1^i^	2.0411 (10)	C4—H4B	0.9800
Co1—N1	2.3071 (12)	C4—H4C	0.9800
Co1—N1^i^	2.3071 (12)	C5—H5A	0.9800
O1—C1	1.2612 (18)	C5—H5B	0.9800
O2—C3	1.2603 (18)	C5—H5C	0.9800
N1—C7^ii^	1.4299 (19)	C6—C6^iii^	1.545 (4)
N1—C7	1.4299 (19)	C6—H6A	0.9900
N1—C6	1.488 (2)	C6—H6B	0.9900
N1—C6^ii^	1.488 (2)	C7—C7^iii^	1.542 (4)
N1—C8	1.523 (2)	C7—H7A	0.9900
N1—C8^ii^	1.523 (2)	C7—H7B	0.9900
C1—C2	1.410 (2)	C8—C8^iii^	1.541 (4)
C1—C5	1.5067 (19)	C8—H8A	0.9900
C2—C3	1.398 (2)	C8—H8B	0.9900
			
O2^i^—Co1—O2	180.0	O1—C1—C5	116.56 (13)
O2^i^—Co1—O1	91.89 (4)	C2—C1—C5	118.50 (14)
O2—Co1—O1	88.11 (4)	C3—C2—C1	124.83 (14)
O2^i^—Co1—O1^i^	88.11 (4)	C3—C2—H2	117.6
O2—Co1—O1^i^	91.89 (4)	C1—C2—H2	117.6
O1—Co1—O1^i^	180.0	O2—C3—C2	125.82 (14)
O2^i^—Co1—N1	90.0	O2—C3—C4	115.39 (13)
O2—Co1—N1	90.0	C2—C3—C4	118.79 (14)
O1—Co1—N1	90.0	C3—C4—H4A	109.5
O1^i^—Co1—N1	90.0	C3—C4—H4B	109.5
O2^i^—Co1—N1^i^	90.0	H4A—C4—H4B	109.5
O2—Co1—N1^i^	90.0	C3—C4—H4C	109.5
O1—Co1—N1^i^	90.0	H4A—C4—H4C	109.5
O1^i^—Co1—N1^i^	90.0	H4B—C4—H4C	109.5
N1—Co1—N1^i^	180.0	C1—C5—H5A	109.5
C1—O1—Co1	128.25 (9)	C1—C5—H5B	109.5
C3—O2—Co1	128.06 (9)	H5A—C5—H5B	109.5
C7^ii^—N1—C7	135.78 (17)	C1—C5—H5C	109.5
C7^ii^—N1—C6	52.41 (12)	H5A—C5—H5C	109.5
C7—N1—C6	109.78 (13)	H5B—C5—H5C	109.5
C7^ii^—N1—C6^ii^	109.78 (13)	N1—C6—C6^iii^	111.15 (9)
C7—N1—C6^ii^	52.41 (12)	N1—C6—H6A	109.4
C6—N1—C6^ii^	137.70 (17)	C6^iii^—C6—H6A	109.4
C7^ii^—N1—C8	55.60 (12)	N1—C6—H6B	109.4
C7—N1—C8	107.38 (12)	C6^iii^—C6—H6B	109.4
C6—N1—C8	105.43 (13)	H6A—C6—H6B	108.0
C6^ii^—N1—C8	58.58 (12)	N1—C7—C7^iii^	112.11 (9)
C7^ii^—N1—C8^ii^	107.38 (12)	N1—C7—H7A	109.2
C7—N1—C8^ii^	55.60 (12)	C7^iii^—C7—H7A	109.2
C6—N1—C8^ii^	58.58 (12)	N1—C7—H7B	109.2
C6^ii^—N1—C8^ii^	105.43 (13)	C7^iii^—C7—H7B	109.2
C8—N1—C8^ii^	138.58 (17)	H7A—C7—H7B	107.9
C7^ii^—N1—Co1	112.11 (9)	N1—C8—C8^iii^	110.71 (8)
C7—N1—Co1	112.11 (9)	N1—C8—H8A	109.5
C6—N1—Co1	111.15 (9)	C8^iii^—C8—H8A	109.5
C6^ii^—N1—Co1	111.15 (9)	N1—C8—H8B	109.5
C8—N1—Co1	110.71 (8)	C8^iii^—C8—H8B	109.5
C8^ii^—N1—Co1	110.71 (8)	H8A—C8—H8B	108.1
O1—C1—C2	124.93 (13)		
			
Co1—O1—C1—C2	0.0	Co1—N1—C6—C6^iii^	179.998 (1)
Co1—O1—C1—C5	180.0	C7^ii^—N1—C7—C7^iii^	−0.002 (1)
O1—C1—C2—C3	0.0	C6—N1—C7—C7^iii^	55.94 (12)
C5—C1—C2—C3	180.0	C6^ii^—N1—C7—C7^iii^	−79.70 (11)
Co1—O2—C3—C2	0.0	C8—N1—C7—C7^iii^	−58.19 (11)
Co1—O2—C3—C4	180.0	C8^ii^—N1—C7—C7^iii^	79.37 (10)
C1—C2—C3—O2	0.0	Co1—N1—C7—C7^iii^	179.998 (1)
C1—C2—C3—C4	180.0	C7^ii^—N1—C8—C8^iii^	−76.77 (11)
C7^ii^—N1—C6—C6^iii^	77.79 (11)	C7—N1—C8—C8^iii^	57.32 (11)
C7—N1—C6—C6^iii^	−55.39 (11)	C6—N1—C8—C8^iii^	−59.70 (11)
C6^ii^—N1—C6—C6^iii^	−0.002 (1)	C6^ii^—N1—C8—C8^iii^	77.23 (10)
C8—N1—C6—C6^iii^	59.99 (10)	C8^ii^—N1—C8—C8^iii^	0.000 (1)
C8^ii^—N1—C6—C6^iii^	−77.99 (10)	Co1—N1—C8—C8^iii^	180.000 (1)

Symmetry codes: (i) −*x*, −*y*, −*z*; (ii) −*x*, −*y*, *z*; (iii) *x*, *y*, −*z*+1.
